# Genome Sequences of Two Grapevine Rupestris Stem Pitting-Associated Virus Variants from Vitis vinifera cv. Riesling in Idaho, USA

**DOI:** 10.1128/mra.01366-22

**Published:** 2023-03-02

**Authors:** Jennifer Dahan, Gardenia E. Orellana, Jungmin Lee, Alexander V. Karasev

**Affiliations:** a Department of Entomology, Plant Pathology, and Nematology, University of Idaho, Moscow, Idaho, USA; b U.S. Department of Agriculture, Agricultural Research Service, Horticultural Crops Production and Genetic Improvement Research Unit, Corvallis, Oregon, USA; DOE Joint Genome Institute

## Abstract

We report the genome sequences of two genetic variants of grapevine rupestris stem pitting-associated virus (GRSPaV) from Idaho, USA. The coding-complete, positive-strand RNA genome of 8,700 nucleotides contains six open reading frames characteristic of foveaviruses. The two Idaho genetic variants belong to GRSPaV phylogroup 1.

## ANNOUNCEMENT

Grapevine rupestris stem pitting-associated virus (GRSPaV) has flexuous filamentous particles and belongs to the genus *Foveavirus* (family *Betaflexiviridae*) (1). GRSPaV is a positive-sense, single-stranded RNA virus with a ~8.7-kb genome spanning six open reading frames (ORFs) (1). The virus exists as a complex of multiple genetic variants, which form four major phylogroups; these genetic variants have been associated with various cultivars of Vitis vinifera L. and its hybrids (1–4). The virus is distributed worldwide and in the United States has been found in grapevines in California, Washington, New York, and Tennessee (1, 5, 6); it has not been reported from Idaho grapevines until now (7–9).

In 2020, six vineyards were sampled in two counties of Idaho (7), for a total of 16 samples. After grinding in liquid nitrogen, total RNA was extracted using the Spectrum Plant total RNA kit (Sigma, St. Louis, MO) and, following ribodepletion using the RiboMinus Plant kit for RNA sequencing (Invitrogen), libraries were prepared using the Kapa RNA HyperPrep kit (Roche) with the NEXTflex unique dual index barcodes set C (Bioo Scientific). After bead-based size selection, the resulting libraries were multiplexed and subjected to high-throughput sequencing (HTS) on a NovaSeq 6000 platform through the University of Idaho Genomics and Bioinformatics Resources Core Facility. A total of 31,500,611 paired-end 250-bp read pairs were produced for the CC04 sample. Raw reads were adapter and quality cleaned using Trimmomatic v0.38 (10) and were mapped against the V. vinifera reference genome using bowtie2 v2.4.4 in local mode (9); unmapped paired-end reads were subjected to assembly using SPAdes v3.15.3 in RNA mode, and analyses using BLASTn and DIAMOND programs were performed with the nonredundant database (11).

**FIG 1:**
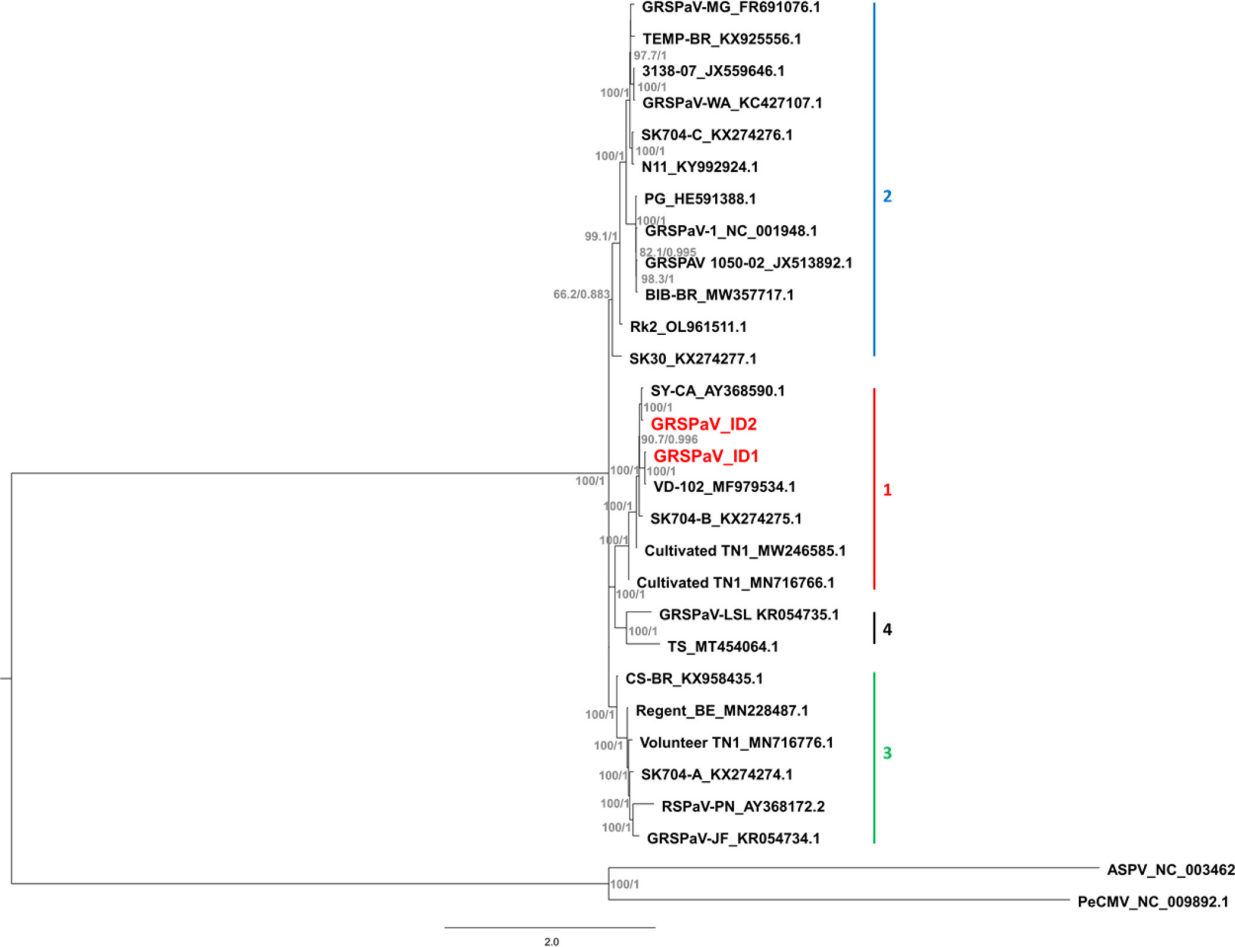
Phylogenetic relationships of the genome sequences of 25 GRSPaV strains from the four main phylogroups of GRSPaV identified by Hily et al. (11) and the GRSPaV sequences obtained in this work, i.e., GRSPaV-ID1 and GRSPaV-ID2. Genome sequences for two other foveaviruses, i.e., apple stem pitting virus (ASPV) and peach chlorotic mottle virus (PeCMV), were added as an outgroup. Nucleotide sequences were aligned with the MAFFT program in Geneious, with default settings in G-insi mode. Phylogenies were inferred using IQ-TREE 2 (13), with ModelFinder for best model fit (14), and branch support was estimated with SH-aLRT/UFBoot (1,000 replicates), as implemented in IQ-TREE 2 (15, 16). The tree was then edited in FigTree v1.4.4.

One of the samples, CC04 from an own-rooted, 38-year-old Riesling vine collected in Nez Perce County, Idaho, yielded a large, 8,700-nucleotide (nt) contig (GRSPaV-ID1) that spanned six ORFs and, based on the BLASTn program (National Center for Biotechnology Information), exhibited 98.5% identity to the GRSPaV isolate 12G412 (GenBank accession number MZ484771) from British Columbia, Canada. The same Riesling sample also yielded three additional contigs of 2,514, 2,893, and 506 nt, which exhibited 98.3%, 98.6%, and 98.8% identity, respectively, to the GRSPaV isolate AMCF clone 3 (GenBank accession number MG938311) from France (12) as determined by BLASTn. Apparently, this sample of Riesling vine contained two distinct genetic variants of GRSPaV. Specific primers (Table 1) were designed to confirm/validate the presence of both GRSPaV variants in the original sample. Using reverse transcription (RT)-PCR as described (9), virus-specific bands were amplified for both GRSPaV genetic variants in the original Riesling vine sample; the specificity of the amplified PCR products was confirmed by Sanger sequencing (9). To extend the genome sequence for the GRSPaV-ID2 variant, additional primers (Table 1) were used in RT-PCR to amplify virus-specific sequences between the three HTS-derived contigs, and the assembly was then verified by remapping the HTS reads to it; the resulting GRSPaV-ID2 contig of 8,481 nt was 92.3% identical to that of GRSPaV-ID1 in a pairwise comparison by BLASTn. The sequenced GRSPaV-ID1 and GRSPaV-ID2 genomes had GC contents of 42.5% and 42.0%, respectively (as determined by Geneious), and were covered by 3,711 and 289 reads, respectively (after assembly with Sanger and HTS contigs). In a phylogenetic analysis (Fig. 1), GRSPaV-ID1 and GRSPaV-ID2 were placed in the same clade 1 designated by Hily et al. (12).

**TABLE 1:** Primers used to validate, confirm, test for the presence, and extend sequences of the GRSPaV isolates from Idaho grapevines, GRSPaV-ID1 and GRSPaV-ID2

Primer name	GRSPaV variant	Genome nucleotide position	Oligonucleotide sequence (5′ to 3′)	Amplicon size (bp)	Intended use^*a*^
GRSPAV1_F1	ID1	2020	CCTGCTCTGGCTAATGATCTG	768	V, C, T
GRSPAV1_R1	ID1	2787	CTTAATCCCGTGAAGGCCCATT		
GRSPAV1_F2	ID1	6693	CACTGAGGACGAGAGTTTCG	916	V, C, T
GRSPAV1_R2	ID1	7608	GACAACCTTGATGATCGCCATC		
GRSPAV2_J1_F	ID2	2481	AGTTTTGACTGTCAACCTGAC	222	V, C, T
GRSPAV2_J1_R	ID2	2702	AAAATTTGGTCATCATCTTCCAG		
GRSPAV2_J2_F	ID2	6808	TCGATGAGTATCTGTCTGTGA	274	V, C, T
GRSPAV2_J2_R	ID2	7081	TTATGCGCAGCTGCCAAATT		
GRSPAV2_J1_F2	ID2	2382	CCGCAATTATTATAACTCATGCT	961	E
GRSPAV2_J1_R2	ID2	3342	GCCTTCAAAGCTAGACCATCT		
GRSPAV2_J3_F	ID2	5787	GCTCATGCAAGCTTTAAAACCT	1,067	E
GRSPAV2_J3_R	ID2	6853	CGTCAAAATCAGAAAAGTCATTC		
GRSPaV2_J4_F	ID2	7522	GAATTCTGGTTCTTGTAGGCGC	690	E
GRSPaV2_J4_R	ID2	8211	ACATGCAAAATCTGCGCAGG		

a V, validate; C, confirm; T, test; E, extend.

### Data availability.

The coding-complete genome sequences of GSPaV-ID1 and GSPaV-ID2 are available in GenBank under accession numbers OQ134941 and OQ134942, respectively. The raw sequence data were deposited in the NCBI Sequence Read Archive (SRA) under BioProject accession number PRJNA931750.
